# Inter-examiner repeatability of measuring phorias using three different methods: Neurolens measurement device, the von Graefe method, and prism cover test

**DOI:** 10.1371/journal.pone.0337126

**Published:** 2025-11-20

**Authors:** Denise Skiadopoulos, Alaina Bandstra, Valerie Kattouf, Corina van de Pol, Vivek Labhishetty, Sumeer Singh

**Affiliations:** 1 Illinois College of Optometry, Chicago, Illinois, United States of America; 2 High Point University School of Optometry, One University Pkwy, High Point, North Carolina, United States of America; 3 Neurolens Inc., Coppell, Texas, United States of America; LV Prasad Eye Institute, INDIA

## Abstract

**Objective:**

Heterophoria is routinely measured during a comprehensive ocular examination. The aim of the current study is to compare the inter-examiner repeatability of the Neurolens measurement device (nMD), a commercially available instrument that objectively assesses phoria, to the inter-examiner repeatability of prism alternating cover test and the von Graefe method.

**Methods:**

91young adults aged between 18–60 years were enrolled. Two experienced optometrists assessed phoria on each subject using three methods: the von Graefe method (VG), prism alternating cover test (PCT) and nMD. VG and PCT were performed at distance (6m) and near (40 cm). The nMD measurements were obtained using virtual distance (6m) and near (50 cm) targets. All the tests were performed in a single session by both the examiners in a randomized order.

**Results:**

All study participants were students, staff, and faculty of the Illinois College of Optometry. Of the 91 participants recruited, 65 were female. All participants completed the study with no missing data. The repeatability analysis showed nMD (distance: 0.69 ± 0.77PD; near: 1.00 ± 0.98PD) to have the smallest mean absolute difference at both distance and near compared to VG (distance: 3.28 ± 3.18PD; near: 4.48 ± 3.99PD) and PCT (distance: 1.50 ± 2.36PD; near: 4.05 ± 3.69PD). Bland Altmann analysis showed that the phoria measurements from nMD exhibited significantly less variability when compared with VG and PCT.

**Conclusions:**

The Neurolens measurement device (nMD) has the highest inter-examiner repeatability when compared to traditional VG and PCT methods. Given that the measurements are objective and repeatable compared to the two traditional methods, this device has the potential to be a useful addition to current methods of clinical practice.

## Introduction

Accurate accommodative and vergence responses are necessary to obtain clear and single binocular vision. Typical clinical measurements to assess the accommodative and vergence systems include the amplitude of accommodation and heterophoria/tropia respectively. Heterophoria is the relative position of the eyes when fusion is disrupted [1]. and is routinely assessed in optometric practice. Clinically, the magnitude of phoria is quantified in prism diopters (PD) and the direction of phoria is specified as exo (outward), eso (inward) or hyper/hypo (vertical). Phoria testing is an important component of the binocular vision evaluation and aids in the diagnosis of conditions such as convergence insufficiency, convergence excess or vertical heterophoria [2–4]. An accurate and repeatable measurement of heterophoria is important when diagnosing and managing binocular vision conditions.

Several methods have been used to measure heterophoria [5–13]. These include the estimated cover test, the prism-neutralized objective cover test, the prism-neutralized cover test with subjective reporting of target movement, von Graefe phorometry with continuous target presentation, von Graefe phorometry with flashed target presentation, the Thorington method, and the Modified Thorington method [7]. Although all the tests measure heterophoria using ocular dissociation, procedures such as the method of dissociation and phoria quantification can be different. Validation studies have quantified the reliability of these tests in primarily two ways, inter-investigator repeatability (across different investigators) and intra-investigator (same investigator) repeatability [7,10]. Both assess the variability in the measurement while the test is performed on the same subject. Based on the existing literature, some of the tests currently used to measure heterophoria have a low level of agreement. Rainey et. al. [7]. evaluated seven different methods of heterophoria measurements: the estimated cover test, the prism-neutralized objective cover test, the prism-neutralized cover test with subjective reporting of target movement, the von Graefe phorometry with continuous target presentation, the von Graefe phorometry with flashed target presentation, the Thorington method, and the modified Thorington method. The study found the von Graefe method to be the least repeatable and the Modified Thorington method to be the most repeatable, with the highest inter-examiner correlation. The prism alternating cover test with subjective reporting of target movement had the second highest correlation [7]. Among all the tests, the prism alternating cover test is one of the most repeatable tests to measure heterophoria. However, most clinical phoria measurements are subjective and rely on either the patient’s response or the doctors’ expertise in identifying dynamic eye movements [14,15].

The Neurolens measurement device (nMD) is a commercially available medical device used to measure phorias. The nMD uses an objective technique to assess eye misalignment using optically set distance and near targets (6 m and 50 cm, respectively). It couples a stereoscopic display with cameras that continuously monitor the patient’s Purkinje reflection (P1) and the pupil. nMD measurements include a pupillary distance measurement, dissociated phoria test, and associated phoria test [16].

Currently there is no published data on the nMD measurement’s repeatability. The aim of this study was to examine the inter-examiner repeatability of the Neurolens measurement device and compare that to the repeatability of the prism alternating cover test and the von Graefe method. The cover test was chosen because it is considered one of the most repeatable tests to measure phoria and is commonly used in the clinics. The von Graefe method, although reported to be less repeatable, is easy to perform and is universally accepted as a standard test to measure subjective phoria [17].

## Methods

Ninety-one optometry school students, faculty and staff were enrolled into the study. Subjects were recruited beginning on August 17^th^, 2023 and ending on October 9^th^, 2023. Sixty-five subjects were female, and twenty-six subjects were male. This study followed the tenets of the Declaration of Helsinki. All study subjects were provided with an informed consent and enrolled only if they were willing to participate and sign the consent form. The study was approved by the Illinois College of Optometry Institutional Review Board (Research Proposal #22028, approved July 10^th^, 2023). Subjects were between 18 and 60 years of age with best corrected distance and near visual acuity of at least 20/30 or better in each eye and refractive error between +/- 6.00 D sphere and not more than –2.00 D of cylinder. Subjects were excluded if they reported a lack of binocular vision or had strabismus. A lack of binocular vision included suppression and/or an inability to view the 250” random dot stereopsis forms. Both spectacle and contact lens wearers were allowed in the study, but for the purpose of consistency, on the day of study visit the participants were asked to wear only their spectacles. Subjects were recruited from among students, staff, and faculty of the Illinois College of Optometry.

The study involved evaluation of the inter-examiner repeatability of the neurolens measurement device, the von Graefe method, and prism corrected cover test in a single visit. Two optometrists highly experienced in binocular vision evaluation (authors AB and VK), served as the examiners. One of the examiners performed the preliminary testing (visual acuities) and then completed phoria testing using each of the three tests in a random order on each subject. After a 5-minute break, the second examiner performed the same phoria tests in a random order. The authors determined that a five minute break between examiners was sufficient, based on an internal trial run. A pre-generated randomization sheet, permuted block design approach created using Microsoft Excel, was used to determine the test and examiner sequence for each subject. Both examiners were masked of the final phoria measurement results to eliminate any bias. For the von Graefe test, subjects were not given any indication of their results and were informed not to discuss anything about the measurements with the second examiner. Subjects wore their habitual spectacles for the prism alternating cover test and the habitual prescription was used in the phoropter for the von Graefe test. Subjects did not need to wear their habitual spectacles while performing the test on the Neurolens measurement device as the device is equipped with a built-in refractive correction feature.

### Prism neutralized cover test distance and near

The prism neutralized cover test was used to determine the amount and direction of each subject’s horizontal phoria. The subjects wore their distance refractive correction for the distance cover test and appropriate near correction for the near test. Subjects fixated on an isolated 20/40 letter at 6 meters and 40 centimeters for the distance and near cover test respectively. The examiner performed at least three cycles of the alternating cover test in primary gaze, occluding for two seconds each time, and observed the eye movements of the subject. If no movement was observed with the initial alternating cover test, base-in and base-out prism were introduced, sequentially, to confirm orthophoria. If reversal occurred immediately at 2 base-in and 2 base-out, the phoria was marked as orthophoria. If reversal did not occur at 2 base-in or 2 base-out, the examiner introduced increasing prism while alternating the cover paddle until reversal was noted. The examiner recorded the prism and base direction as the highest amount of prism prior to reversal. If movement was observed with the initial alternating cover test, the examiner neutralized the amount of heterophoria with a prism bar by introducing base-in prism to neutralize exophoria or base-out to neutralize esophoria while alternating the cover paddle. The examiner recorded the prism and base direction as the highest amount of prism prior to reversal of the eye movement.

### von graefe dissociated lateral phoria test distance and near

The von Graefe dissociated lateral phoria test was used to determine the amount and direction of each subject’s phoria using a phoropter with the subject’s prescription in place. The subject’s eyes were dissociated using 12 PD base-in over the right eye and 6 PD base-up over the left eye, as the subject viewed a 20/40 block of letters. If appropriate dissociation was not achieved with 12 PD base-in, the base-in prism was increased until such dissociation was achieved. As the subject fixated on the bottom block of letters and was instructed to keep the letters in that block clear, the amount of horizontal prism was decreased until the subject reported that the blocks were vertically aligned. The examiner passed the endpoint and then increased the base-in prism until the subject again reported that the letters were vertically aligned. The two endpoints were averaged, and the examiner recorded the prism and base direction for both the distance (6m) and near (40 cm) test.

### Neurolens measurement device (nMD)

**Fig 1:**
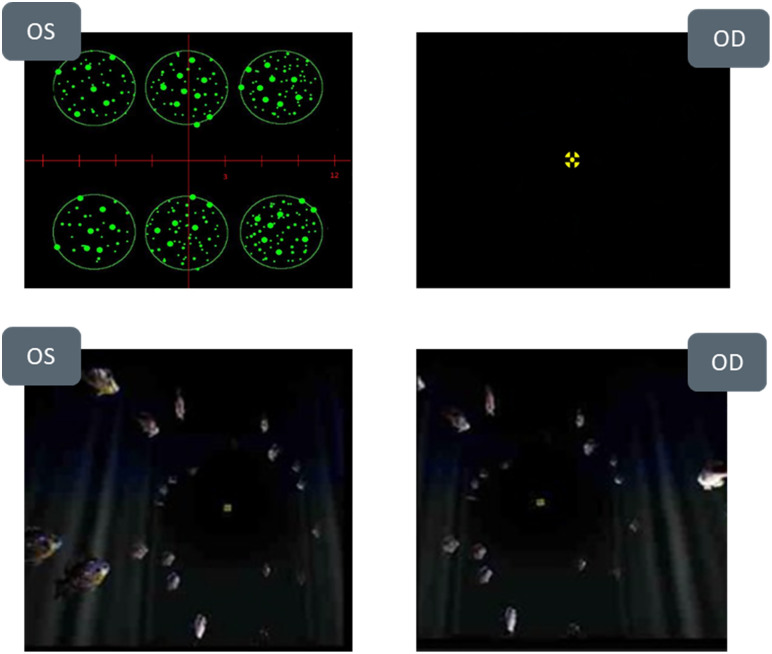
Top images: Patient view during nMD Base Alignment (yellow fixation cross viewed by OD, Petri dishes viewed by OS). Bottom images: Patient view during nMD Fine Alignment. The target alternates display between OD and OS at different positions until the eye movement is neutralized.

nMD employs an objective technique to assess eye misalignment at distance (6 m) and near (50 cm) in primary gaze. The device uses two displays to present fixation and other stimuli separately to each eye, while dual eye trackers (60 Hz) monitor eye movements by tracking patient’s Purkinje reflection (P1). After calibrating with a 60 mm pupillary distance chart mounted on to the device, the optical targets are aligned to each participant’s pupil center. The fixation target is then placed at the vergence distance, establishing a reference gaze angle. Eye movements are tracked as deviations from this origin. Both device displays can be moved along the optical axis behind the imaging optics. By physically adjusting the display positions, the object distance is changed. The device can change the refractive power needed to bring the target to focus at 6 m and 50 cm. Using moving displays, the device both corrects for the spherical equivalent of the patient’s refractive error (Hyperopia up to +6.00D and myopia up to –9.0D) and places the image at either 6 m or 50 cm depending on the stage of the measurement. The device is capable of measuring phorias up to 20 prism diopters of misalignment. The test consists of a central fixation target (yellow cross) that subtends 0.6 degrees of visual angle at both distance and near and an actively moving peripheral target (fish, Fig 1). If the participant is uncooperative or if eyelids obstruct the Purkinje image, the device halts the test and notifies the examiner to restart. Clinicians can monitor eye movements in real-time via an externally mounted screen on the advice and advise the patient to fixate properly in cases of eccentric fixation. The device can measure phorias up to 20 prism diopters of misalignment. During this study the subject was instructed to fixate on the central fixation cross throughout the measurement. The measurement process included three measurement steps: pupillary distance measurement, dissociated phoria, and associated phoria.

1) The subject’s PD was initially measured. The fixation cross displays were adjusted by the instrument to match with the subject’s pupil center. At the end of this step, the center of the display’s coincided with the pupil center for each eye.
2) The instrument next measured the dissociated phoria. Central and peripheral targets that could be fused were presented at each distance based on the subject’s measured PD. The eyes were then dissociated using non-fusible targets (fixation cross in one eye and a ruler with 6 petri-dishes in the other eye, Fig 1). The direction of gaze was measured after the eyes were stabilized for a set period of time. This step is called the base alignment measurement.
3) Fusible targets were then placed in the periphery at the gaze position measured during the dissociated phoria measurement, serving as the peripheral fusion lock. The central fixation cross was flashed to one eye at a time, and the geometric position of the yellow cross was adjusted until the eye movement (if any) was neutralized. In other words, the Neurolens device performed a central associated phoria test after neutralizing the dissociated phoria. This step is called the fine alignment measurement.

The test duration for the nMD was approximately two minutes per subject. The final output screen provided the patient’s horizontal and vertical phoria measurements. Of note, the test duration for the entrance testing and all three phoria measurements by both examiners, including the break, took an average of twenty minutes.

### Statistical methods

The primary endpoint was to compare the inter-examiner difference in the nMD measurements to the measurements obtained with prism neutralized cover test and the von Graefe method. Statistical analyses were performed using SPSS 24 (IBM Corp., Armonk, New York, USA) and GraphPad Prism (Boston, USA). No formal sample size calculation was performed; however, a sample size of 90 participants was deemed feasible for this study.

Exophorias were represented with a negative sign and esophorias with a positive sign. When evaluating the mean phoria measurements, given the two sign conventions, there was a possibility that the eso and exo deviations would negate one another. This could bias the results and mask the true differences between the examiners. To evaluate the true difference between the examiners, the mean absolute differences in the heterophoria between the two examiners was plotted. Data normality was assessed using the Shapiro–Wilk test. To evaluate the inter-examiner repeatability, Bland Altmann plots (mean bias and standard) and Intraclass Correlations (Pearson correlation coefficient) were used. In a Bland-Altman plot, bias represents the average difference between two measurement methods, and the standard deviation indicates the variability of these differences. Spearman correlation was considered poor if r was 0.3 or less, modest if between 0.3 and 0.7, and good if 0.7 or higher.

## Results

Table 1 represents the mean and standard deviation of the phoria measurements obtained using prism cover test, the von Graefe method, and Neurolens measurement device at distance and near for both the examiners. Negative sign indicates exophoria. Additionally, no participants exhibited signs of fatigue or withdrew early from the study.

**Table 1:** Mean ± SD of the phoria measurements using each test for each examiner.

Test	EXAMINER I	EXAMINER II
Prism Cover Test – Distance (PD)	−1.07 ± 2.82	−1.13 ± 2.64
von Graefe – Distance (PD)	−1.14 ± 4.06	−0.63 ± 3.87
Neurolens – Distance (PD)	−1.81 ± 1.85	−1.58 ± 1.86
Prism Cover Test – Near (PD)	−3.63 ± 6.26	−4.00 ± 5.68
von Graefe – Near (PD)	−4.82 ± 6.80	−5.89 ± 5.99
Neurolens – Near (PD)	−4.67 ± 3.37	−4.34 ± 3.42

### Inter-examiner repeatability

**Fig 2:**
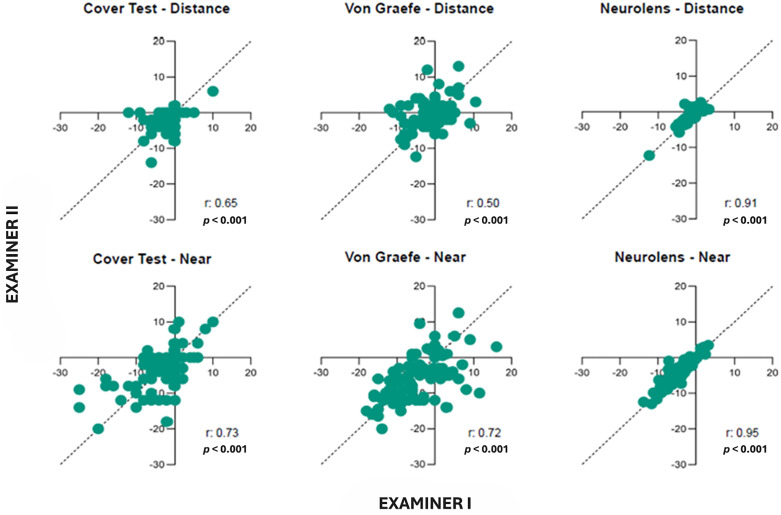
Correlation plots between the two examiners. The dotted line indicates the 1:1 line. Any points on this line would indicate that both examiners reported the same phoria measurement. The top row indicates distance measurements and bottom row indicates near measurements for each test. ‘r’ value indicates the correlation coefficient. Negative value indicates exophoria and positive value indicates esophoria.

Inter-examiner correlations for each test at both distance and near were performed and the correlation plots are shown in Fig 2. Neurolens measurement device was found to have the highest degree of correlation (r = > 0.90, *p* < 0.001) between the two examiners at both distance and near.

**Fig 3:**
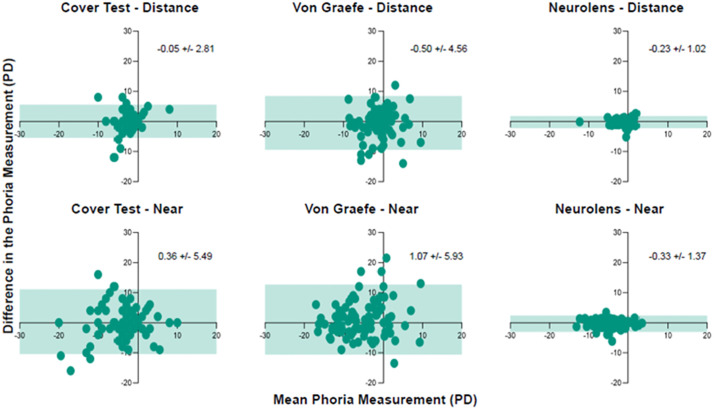
Bland-Altmann Plots. The difference in the phoria measurement between the two examiners were plotted as a function of the mean phoria measurement. The shaded region indicates the 95% limits of agreement, i.e., 1.96 times the SD of the differences in the measurement. The numbers on each plot indicate the mean difference in the phoria measurement between the two examiners and the standard deviation (SD).

**Fig 4:**
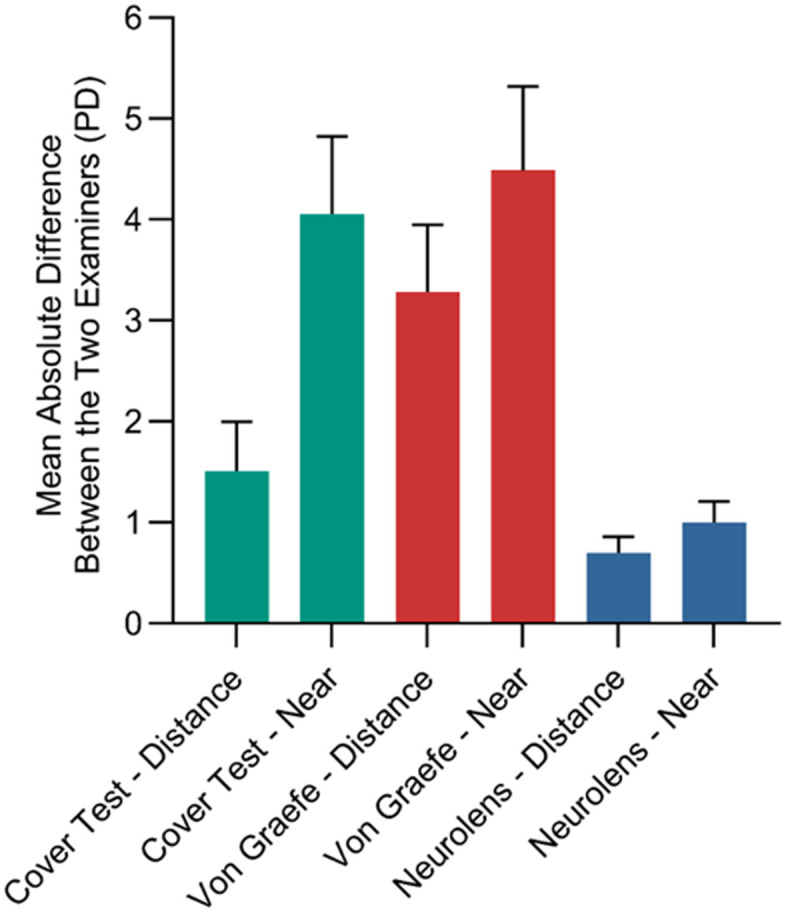
Absolute mean difference between the two examiners (PD) was plotted for each test at both distance and near. Error bars indicate the 95% confidence intervals.

Results of the Bland-Altmann plots are shown in Fig 3. As shown in Fig 3, Neurolens measurement had the least measurement variability compared to the other two tests. Fig 4 shows the absolute mean difference between the two examiners along with the standard error for all the three tests at both distance and near.

### Examiner(s) correlation of study procedure outcomes

**Fig 5:**
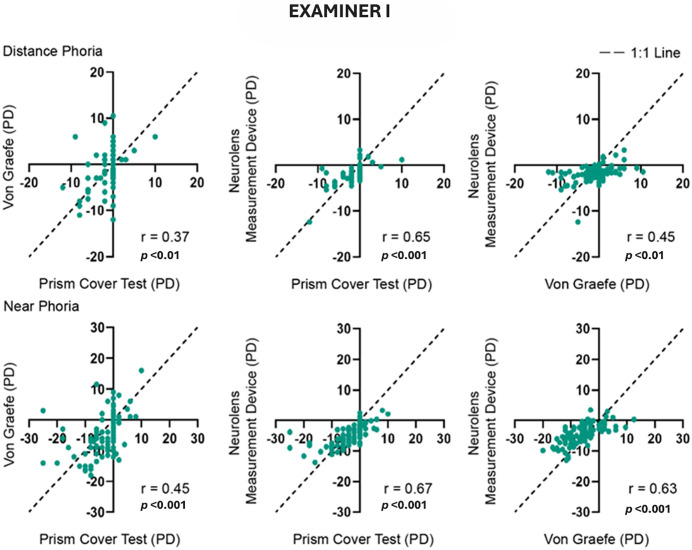
Correlation between the three tests performed by Examiner I. The dotted line indicates the 1:1 line. The top row shows comparisons between distance measurements and bottom row between near measurements. ‘r’ value indicates the Pearson correlation coefficient. Negative values indicate exophoria and positive values indicate esophoria.

**Fig 6:**
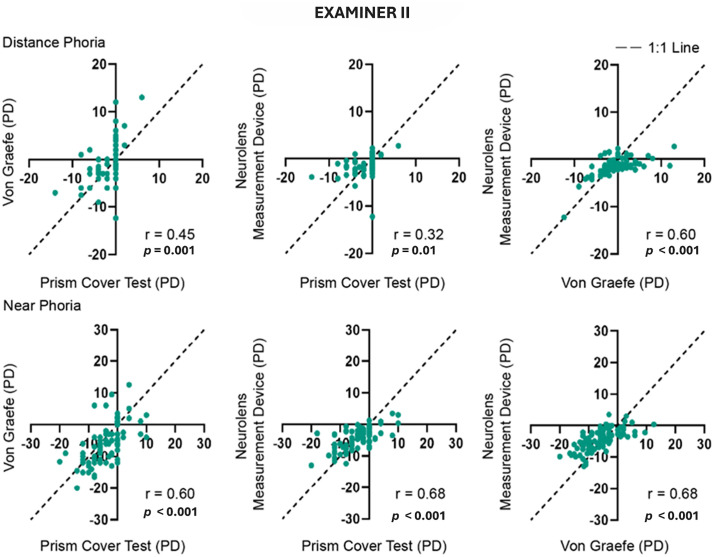
Correlation between the three tests performed by Examiner II. The dotted line indicates the 1:1 line. The top and bottom rows show comparisons between distance and near measurements respectively. Negative values indicate exophoria, and positive values indicate esophoria. ‘r’ value indicates Pearson correlation coefficient.

Test-Test correlation at both distance and near was done between all the tests being compared in the study. These comparisons were done for both Examiner I (Fig 5) and Examiner II (Fig 6). Overall, good correlations were found between the three tests and the correlations were better at near compared to distance. Cover Test and the von Graefe method showed modestly better correlations with Neurolens measurement device compared to a correlation between themselves.

Results for Bland-Altmann plots are shown in Table 2. Overall, both the von Graefe method and Neurolens measurement device showed an exophoric bias in the phoria measurements compared to Cover Test at both distance and near. Neurolens measurement was more exophoric with distance measurements compared to von Graefe measurements. However at near, von Graefe measurements were more exophoric than Neurolens and cover test measurements.

**Table 2:** Bland Altmann analysis between different tests employed in the study at both distance and near. These comparisons were done separately for examiner I and II. A positive value indicates an exophoric bias of the second test compared to the first test. A negative value indicates an esophoric bias of the second test compared to the first test.

Bland-Altmann Analysis – First Examiner			
Clinical Tests (First test, Second test)	Sample size (N)	Mean Difference	Standard Deviation
Distance Phoria Measurements			
Cover Test & von Graefe	91	0.06	4.00
Cover Test & Neurolens Device	91	0.75	2.18
von Graefe & Neurolens Device	91	0.68	3.62
Near Phoria Measurements			
Cover Test & von Graefe	91	1.18	6.84
Cover Test & Neurolens Device	91	1.04	4.70
von Graefe & Neurolens Device	91	−0.14	5.34
**Bland-Altmann Analysis – Second Examiner**			
**Clinical Tests**	**Sample size (N)**	**Mean Difference**	**Standard Deviation**
Distance Phoria Measurements			
Cover Test & von Graefe	91	0.49	3.54
Cover Test & Neurolens Device	91	0.45	2.68
von Graefe & Neurolens Device	91	0.94	3.11
Near Phoria Measurements			
Cover Test & von Graefe	91	1.89	5.18
Cover Test & Neurolens Device	91	0.39	4.18
von Graefe & Neurolens Device	91	−1.49	4.45

## Discussion

The current study evaluated the inter-examiner repeatability of the Neurolens measurement device and compared it to standard clinical tests which measure heterophoria. The Neurolens measurement device was the most repeatable test among the three tests compared in the current study. Both Bland-Altman and correlation analysis validated the inter-examiner repeatability of the Neurolens measurement. The subjective prism cover test was the second most repeatable test and the von Graefe method had the least repeatability. The mean absolute error analysis demonstrated a similar result: the Neurolens measurement exhibited less than 1PD difference between the two examiners while the other two tests had a mean absolute error that was significantly higher (near> distance). Objective testing may be less variable than the subjective findings amongst two different examiners, and the data supported that. In terms of the test-test accuracy, modest correlations were noted between the three tests with correlations being better at near compared to distance measurements.

Prior to this study, inter-examiner repeatability of the Neurolens measurement had not been assessed. The inter- and intra-examiner repeatability of prism cover test has been validated previously [10,18,19]. The results of the current study regarding the variability of the prism cover test are consistent with the previous studies [20]. The inter- and intra-examiner repeatability of the von Graefe test has also been previously validated [4,11–13, 7]. In agreement with the previous studies, the von Graefe method was the least repeatable test among the three tests evaluated in the current study. Previous studies have explored the validity of an objective way of assessing heterophoria and have reported that the objective estimation was significantly more repeatable than the standard subjective tests such as cover test [5,8]. These studies concluded that using calibrated eye trackers significantly improved the variability of the heterophoria measurement when compared to subjective tests. The data in the current study on the objective Neurolens measurement compared to the subjective tests correlates with the conclusions of these previous studies.

### Subjective nature of the tests

Phoria tests can yield variable measurements since the dynamics of an individual’s eye movement under dissociated conditions can vary [5]. With subjective tests, the time point at which a patient responds or at which the doctor measures phoria could impact the accuracy of the phoria estimation. Also, it can be difficult for the novice practitioner to accurately identify small eye deviations which may lead to a misdiagnosis or underestimation of a binocular vision issue [15,21]. This is especially crucial because the magnitude of phoria may not always correlate with the symptomology [3]. Therefore, small ocular deviations that could cause symptoms need to be identified accurately so an appropriate treatment is provided.

Given the subjectivity of the tests, examiner expertise in accurately identifying the eye misalignment could lead to variability in the estimation of the phoria [18,22]. Further, the standard employed to define the neutralization point in tests such as prism cover test could lead to inter-examiner variability in the estimation of phoria [23]. Overall, the limitations of the currently employed tests could be addressed by addition of an objective technique that does not rely on either the patient’s response or the doctors’ judgement [8].

### True orthophoria vs measurement error with Cover Test

The prism cover test is considered as one of the most repeatable tests and the current study found it to be better than the von Graefe test. It is, however, subjective and has two versions: either examiner-reported, or patient-reported phoria estimation method. The current study employed an examiner-reported measurement protocol. Previous studies reported that the ability of the observer (examiner) to detect small misalignments is limited and reported that anything smaller than 2PD could be overlooked [14,15, 10]. In agreement, the current study found that examiners missed more eye deviations with a cover test compared to the von Graefe method and the Neurolens measurement.

#### Examiner 1.

For distance measurements, 59 out of the 91 (65%) cover test measurements were recorded as ‘0’ heterophoria or orthophoria using a cover test. 76% and 44% of these measurements were measured to have a phoria greater than 1PD with the Neurolens and von Graefe test respectively. 32% and 40% of these measurements were measured to have a phoria greater than 2PD with the Neurolens and von Graefe test. For near measurements, 28 out of the 91 (30%) measurements were recorded as ‘0’ heterophoria using a cover test. 89% and 78% of these measurements were measured to have a phoria greater than 1PD with the Neurolens and von Graefe test. 71% and 68% of these measurements were measured to have a phoria greater than 2PD with the Neurolens and von Graefe test.

#### Examiner 2.

For distance measurements, 63 out of the 91 (69%) cover test measurements were recorded as ‘0’ heterophoria using a cover test. 82% and 60% of these measurements were measured to have a phoria greater than 1PD with the Neurolens and von Graefe test. 25% and 58% of these measurements were measured to have a phoria greater than 2PD with the Neurolens and von Graefe test. For near measurements, 24 out of the 91 (26%) measurements were recorded as ‘0’ heterophoria using a cover test. 79% of these measurements were measured to have a phoria greater than 1PD with both the Neurolens and von Graefe test. 62% and 70% of these measurements were measured to have a phoria greater than 2PD with the Neurolens and von Graefe test.

The current study results suggest that a subjective test such as a cover test, which relies on the examiner to detect small and dynamic eye movements, could miss smaller phorias. The optometrists in this study were highly experienced in binocular vision testing, and the number of missed deviations could be larger with clinicians who do not perform binocular vision testing regularly. While it is unlikely that a small phoria would cause significant symptoms in most patients, objective measurements that detect small phorias could be beneficial for some patients, such as those who have suffered a traumatic brain injury.

### Study limitations

The most significant limitation of this study is that it only assesses inter-examiner repeatability, not the validity or reliability of each test, and not intra-examiner comparisons between tests. Future studies on these topics, particularly evaluating the accuracy of the nMD to existing associated and dissociated phoria measurements, would certainly be of value to clinicians. However, this particular study was only designed to measure inter-examiner repeatability of each device.

Another limitation is that unlike the traditional methods where the participants wore full spectacle correction, the nMD optically creates the subject’s spherical equivalent rather than sphere and cylinder separately, therefore subjects with cylinder greater than 2 diopters were excluded. This may have resulted in a blurry view of the target for certain subjects, potentially affecting the accommodation and vergence responses. The inter-examiner repeatability of the nMD may therefore be different if patients with higher astigmatism are tested. This study did not include vertical phoria measurements, which limits the data presented.

The tests chosen could be a limitation of the study. There are many other tests that measure heterophoria, such as Modified Thorington. The investigators chose to focus on the von Graefe method and cover test since they are, by observation, frequently used at the Illinois College of Optometry clinic. One might ask why the von Graefe method would be frequently used in an academic optometric setting given its low repeatability. Binocular vision faculty at the college have noted that it is helpful to teach students both in-phoropter and out-of-phoropter methods of assessing phoria, given that the phoropter may induce proximal convergence and affect measurements [24]. Therefore the von Graefe method is the in-phoropter test of choice. Further investigation into inter-repeatability of the Neurolens compared to other clinical phoria tests, such as the Modified Thorington method, could be beneficial to clinicians and optometry students.

Finally, the difference in the placement of the near targets could have affected the study results. Cover test and the von Graefe method are performed at 40 cm, and nMD is performed with targets placed virtually at 50 cm. This additional 10 cm, in addition to proximal convergence induced by the instrument, may introduce biases in the nMD results and should be considered when comparing to measurements taken at 40 cm.

## Conclusion

The current study evaluated the inter-examiner repeatability of the Neurolens measurement device and compared that to the two commonly employed tests to measure heterophoria, subjective prism cover test and the von Graefe method. Both Bland Altmann and correlation analysis showed that the objective Neurolens measurement device exhibited a significantly higher inter-examiner repeatability compared to the other two tests. Within the sample tested, the objective nature of the nMD appeared to improve the variability in the phoria estimation compared to the traditional subjective tests. In practice, cover test and the von Graefe method are well-established, valid tests that have significant clinical value, including the ability to make qualitative observations and perform testing at the working distance of most patients. However repeatable phoria measurement technology may be a beneficial addition to the current battery of binocular vision testing.

## Supporting information

S1 Data Full raw data set. (XLSX)
